# Concerning Contests

**Published:** 1887-09

**Authors:** 


### Editorial.

### CONCERNING CONTESTS.

It is with regret that we see in *The Dental Headlight* correspondence between a dentist of Atlanta, Ga., and Dr. E. Parmly Brown, concerning the Herbst method of filling teeth. We are of the opinion that Dr. Brown's letter will convey an impression which its author scarcely intended. Dr. Herbst did not come to America in a spirit of bravado, nor to enter into a contest with any one as to his relative skill as an operator, or even as to the relative merits of the method of introducing gold which goes by his name. During his whole visit here he exhibited a becoming modesty of demeanor, and an anxiety to learn from our best exemplars, which gained him many admirers. At his very first appearance before a body of dentists, he explicitly said that he came to submit the process which he had devised, and which was the child of his necessities and not of his personal ambition, to the criticisms of those whom in all the world he thought best qualified to judge of its merits. He did not offer it as a substitute for old and approved methods, but as a coadjutor. It is not a succedaneum, but an auxiliary. Freely did he give his time, and unweariedly did he expend his energies, without ever receiving or attempting to obtain one cent for his public or private clinics. Who among us can boast such a truly professional spirit ? What American dentist has visited Europe with such unselfish aims, or has left such a record as the result of his visit ?

Dr. Bödecker's name has been, very much to his regret, we are certain, introduced into the discussion. We have had personal knowledge of the great sacrifices of time and money made by Dr. Bödecker, with the sole desire to benefit dentistry and his brother dentists. To our knowledge he has never spoken, but others have computed that he must have expended at least $3,500 in money, besides much more in time, with no thought whatever of reaping any personal benefit from it. He did it because he loved his profession and his professional brethren, and because he believed that the so-called Herbst system was fraught with great good to dentistry. He still believes it, and who dare question either his intelligence or honesty ? Clinics were given all over the country. Who do dentists imagine paid the expenses of the trips, and furnished the gold and material in most of the cases? Dr. Herbst is a poor man, who all his life-time has traveled but a rough road, and it was beyond his power to give more than his time.

After all the unselfish labors of Herbst, after all the sacrifices of Bödecker, and the generous and unselfish treatment which both extended toward other operators who antagonized their benevolent efforts, it does seem a little cruel that they should be placed in an attitude so foreign to that which they of themselves would assume. We are not personally engaged in the practice of the Herbst method, but we feel that we owe a great weight of gratitude to the men who labored so hard for our good, in common with that of others, and may our hand be palsied ere it writes a word that shall express anything but appreciation of the debt.

That Herbst's visit to America resulted in a general quickening of dental intelligence and a broadening of our practical views, few will care to dispute. Good dentistry does not exclusively consist in the ability to punch a definite amount of gold into a special-sized hole. There is a broader basis than mere mechanical skill, upon which dentistry, as a profession, must rest. There is a professional modesty and courtesy, an ethical spirit and tone which must exist if we are to be numbered among the liberal professions. What matters it to dentistry which of her favorite sons, Brown, or Webb, or Abbott, or Bödecker, could pack the most gold into a glass tube? What does the average dentist care about this question of personality? Good practice means something infinitely above and beyond that. There is a principle involved, and for the maintenance of that we are all supposed to be earnestly laboring. Away with the idea of a personal contest for superiority! We must take broader views than this if we are ambitious of any real good. If our names are to be inscribed upon the roll of honor, we must not forget that we cannot segregate our own professional interests from those of our brethren, without being the sufferers.

We have made this correspondence the text for a little editorial sermon, not that we may adorn a tale, for, as was stated at the outset, we have no idea that Dr. Brown intended or conceived that a construction which his letter will easily bear would be placed upon it, but that we may point a moral which all of us at times need seriously to ponder over, Dr. Brown no more than the rest of us.

